# A modified A* algorithm combining remote sensing technique to collect representative samples from unmanned surface vehicles

**DOI:** 10.3389/fnbot.2024.1488337

**Published:** 2024-10-22

**Authors:** Lei Wang, Danping Liu, Jun Wang

**Affiliations:** School of Advanced Manufacturing Engineering, Hefei University, Hefei, China

**Keywords:** unmanned surface vehicles, A* algorithm, remote sensing technique, water sampling path planning, sampling representativeness, path length, chlorophyll-a

## Abstract

Ensuring representativeness of collected samples is the most critical requirement of water sampling. Unmanned surface vehicles (USVs) have been widely adopted in water sampling, but current USV sampling path planning tend to overemphasize path optimization, neglecting the representative samples collection. This study proposed a modified A* algorithm that combined remote sensing technique while considering both path length and the representativeness of collected samples. Water quality parameters were initially retrieved using satellite remote sensing imagery and a deep belief network model, with the parameter value incorporated as coefficient *Q* in the heuristic function of A* algorithm. The adjustment coefficient *k* was then introduced into the coefficient *Q* to optimize the trade-off between sampling representativeness and path length. To evaluate the effectiveness of this algorithm, Chlorophyll-a concentration (Chl-a) was employed as the test parameter, with Chaohu Lake as the study area. Results showed that the algorithm was effective in collecting more representative samples in real-world conditions. As the coefficient *k* increased, the representativeness of collected samples enhanced, indicated by the Chl-a closely approximating the overall mean Chl-a and exhibiting a gradient distribution. This enhancement was also associated with increased path length. This study is significant in USV water sampling and water environment protection.

## 1 Introduction

Water pollution has long been a critical global concern (Schwarzenbach et al., 2010). Daily water safety has a direct and perpetual impact on human beings (Bhagwat, 2019). Water sampling and quality testing are essential components for safeguarding the water environment (Zhang, 2024; Behmel et al., 2016). Traditional water sampling methods primarily involve manual collection, which is time-consuming and labor-intensive (Pule et al., 2017). In recent years, the innovation of unmanned surface vehicles (USVs) has resulted in their widespread use in water sampling, effectively replacing traditional manual sampling methods (MahmoudZadeh et al., 2022). This advancement facilitates efficient and flexible water sample collection and environmental monitoring of aquatic ecosystems (Yu et al., 2022).

The initial USV-based sampling approach is typically collecting samples after reaching a designated water area through remote manipulation (Yu et al., 2019). While this technique is simple, effective control in vast water areas is challenging due to restrictions in observation and communication distance (Young and Peschel, 2020; Peng et al., 2021). Subsequently, studies on path planning algorithms for USV sampling gradually emerged (Yu et al., 2022; Song et al., 2019; Liu and Bucknall, 2015), and USVs can navigate autonomously along the planned routes. Regardless of global, local or hybrid path planning algorithms, they all focus on the optimization of path length, time cost, smoothness, and collision avoidance (Xing et al., 2023; Yu et al., 2022; Vagale et al., 2021; Yu et al., 2019; Yu et al., 2024). However, these studies tend to overemphasize path optimization, neglecting the core objective of collecting representative samples that accurately reflect the condition of the water body.

In an USV water sampling mission, multi-goal path planning is another prevalent path planning algorithm (Ma et al., 2021; Luo et al., 2024). For example, USVs are employed to collect data and samples from multiple dispersed water quality monitoring stations (Liu and Bucknall, 2018). Unlike the aforementioned path planning algorithm that features a single goal point, the multi-goal path planning algorithm has multiple goal points, requiring the algorithm to design a route that efficiently visit each goal point with minimal cost (Liu and Bucknall, 2018). The task allocation in the multi-goal path planning can be summarized as a Travelling Salesman Problem. This type of algorithm is typically suitable for situations where the sampling points are predetermined, such as in the collection of samples from multiple monitoring stations or designated locations. Although samples collected by this method may be representative, its practicality is restricted and it is not suitable for situations where the sampling site is not predetermined. Therefore, there is an urgent requirement for an algorithm that meets the three crucial needs: minimizing cost, ensuring collecting representative samples, and adapting to situations where sampling points are not predetermined.

The A* algorithm is a well-known global path planning algorithm used to find the shortest route in a known global map. As a heuristic search algorithm, A* is more efficient than other deterministic methods due to its ability to consistently provide an optimal path with the minimum cost, thereby ensuring its completeness (Song et al., 2019). A* algorithm is widely used in the field of USV path planning due to its simplicity and high efficiency. It was initially introduced to this field by integrating with reactive navigation to propose an enhanced three-layered architecture for USV path planning within a harbor (Casalino et al., 2009). More studies were conducted on the basis of A* algorithm to adapt to the path planning in specific situations (Song et al., 2019; Sang et al., 2021; Singh et al., 2018; Casalino et al., 2009; Naeem et al., 2012). For instance, Sang et al. (2021) proposed a hybrid path planning algorithm that combines A* and artificial potential field to solve the path planning problem of USV formations. Singh et al. (2018) proposed a constrained A* algorithm to address the motion planning problem for USV navigating in a maritime environment containing dynamic obstacles.

Remote sensing refers to the science of identifying Earth’s surface features and estimating their geo-biophysical properties through the use of electromagnetic radiation as a medium of interaction (Campbell and Wynne, 2011). With the advancement of remote sensing technology, it is now possible to obtain concentrations of water quality parameters in a non-contact manner, over large areas, and in quasi-real time (Ritchie et al., 2003; Wang and Yang, 2019; Gholizadeh et al., 2016). The concentrations of water quality parameters retrieved from remote sensing imagery can serve as prior knowledge for selecting sampling locations to address the representativeness of sampling points.

In this paper, a modified A* algorithm combining remote sensing technique was proposed, which simultaneously minimizes cost while ensuring that the sampling points are representative and not predetermined. Chlorophyll-a concentration (Chl-a), an important indicator of aquatic ecological status and can reflect the eutrophication level of the water body, was employed as the test water parameter to validate the proposed algorithm. Chaohu Lake was selected as the study area. The main contributions of this work are as follows:

1. To ensure the representativeness of collected samples, the heuristic function of the A* algorithm was modified by incorporating a sampling coefficient *Q*. The coefficient *Q* was derived from the Chl-a value retrieved by remote sensing technique through a negative exponential transformation of *e*. This adjustment ensured that Chl-a of the collected samples approximated the overall mean Chl-a of the entire water body and exhibited a gradient distribution.
2. To optimize the trade-off between sampling representativeness and path length, an adjustment coefficient *k* was introduced into the calculation formula of the sampling coefficient *Q*. As coefficient *k* increases, the modified A* algorithm plays less emphasis on path length but more emphasis on the Chl-a’s spatial distribution. More representative samples will be collected at the expense of increasing path length, and vice versa.

## 2 Study area and materials

### 2.1 Overview of study area

**Figure 1:**
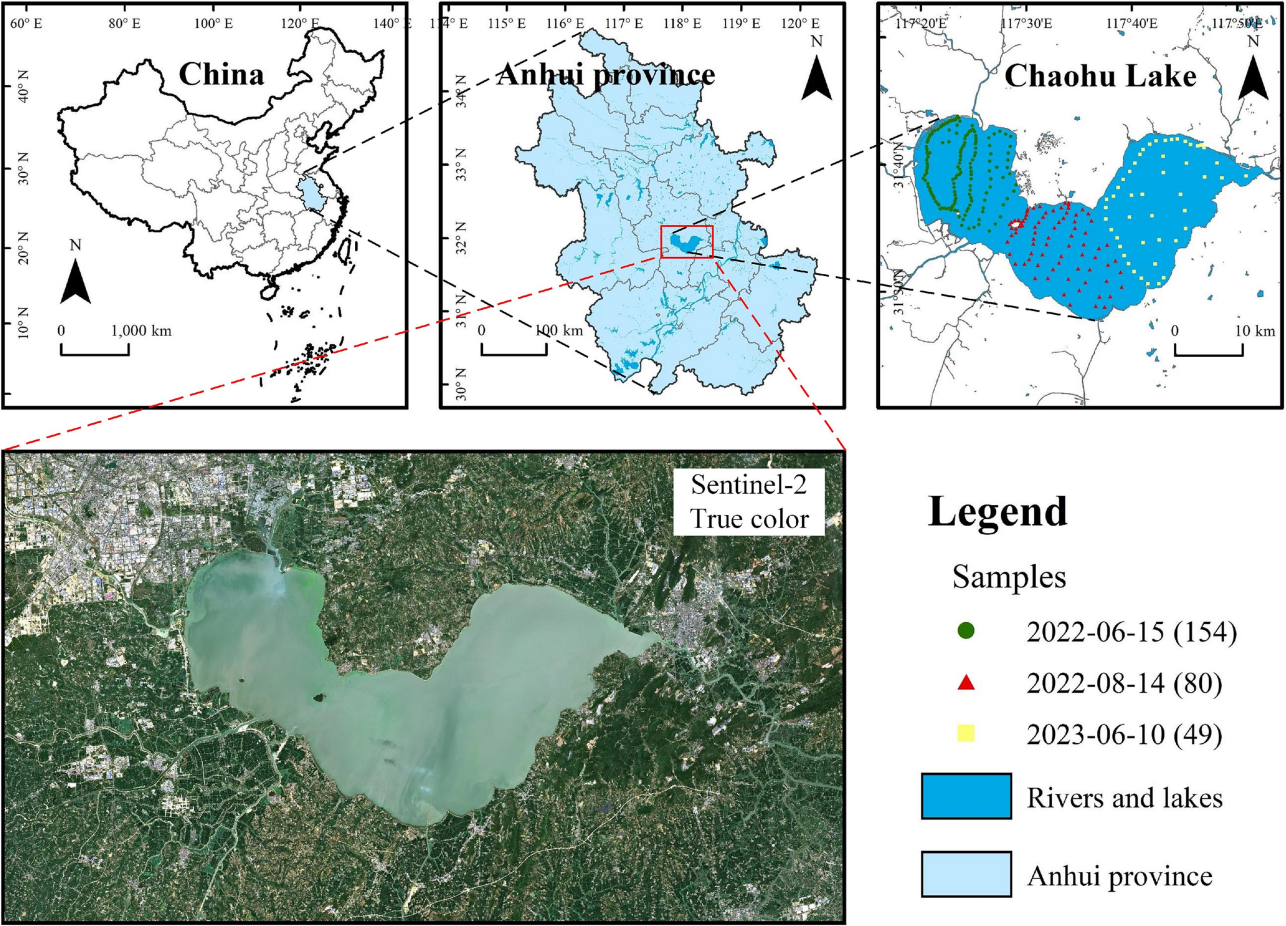
Location and true-color image of the study area, Chaohu Lake. The sample points in different colors indicate samples collected on different dates, while the numbers in brackets represent the quantity of sample points.

Chaohu Lake, situated in central Anhui Province, is one of China’s five prominent freshwater lakes (Figure 1). Covering approximately 770 km^2^, the lake maintains an average depth of 2.7 m and an average water level of 8.0 m (Fang et al., 2019). Chaohu Lake receives inflow from approximately 33 rivers, with 8 primary tributaries including Nanfei River, Shiwuli River, Pai River, Hangbu River, Zhegao River, Shuangqiao River, Zhao River, and Baishitian River, and its principal outflow is the Yuxi River (Wang and Dou, 1998). The annual mean temperature in the Chaohu Lake basin is approximately 15 ~ 16°C, and the average annual rainfall varies between 500 and 1800 mm (Yang et al., 2020). The escalating population within the basin, coupled with the swift advancement of industrial and agricultural activities, has resulted in the discharge of substantial amounts of industrial effluents and municipal wastewater into the lake. This influx has notably heightened the levels of nutrients and organic substances and accelerated the process of lake eutrophication (Yang et al., 2013).

### 2.2 Materials

Samples in Chaohu Lake were collected from a manned vessel under clear weather conditions coinciding with the transit of Sentinel-2 satellites on June 15, 2022, August 14, 2022, and June 10, 2023. A total of 154, 80, and 49 samples were acquired on these respective dates, with the sampling sites delineated in Figure 1. At each sampling point, 1,000 mL of surface water samples were collected, and Chl-a was subsequently determined using spectrophotometric method once the samples were transported back to the laboratory. For all sampling points, the highest Chl-a was recorded on June 10, 2023, at the northeastern shoreline of Chaohu Lake, reaching 192.78 mg/m^3^. The lowest value was observed on June 15, 2022, at the western part of the lake, at 23.25 mg/m^3^. The average Chl-a across the sampling sites was 65.43 mg/m^3^, indicating a significant level of eutrophication in Chaohu Lake.

The harmonized Sentinel-2 Multispectral Instrument (MSI) Level-2A surface reflectance datasets from the aforementioned 3 days were gathered for retrieving Chl-a. The MSI dataset was downloaded from the Google Earth Engine platform,^1^ with the cloud coverage remaining below 10%. Eleven bands from these images were selected, encompassing four 10-m resolution bands: 496 nm (B2), 560 nm (B3), 665 nm (B4) and 835 nm (B8), and six 20-m resolution bands: 704 nm (B5), 740 nm (B6), 782 nm (B7), 864 nm (B8A), 1,613 nm (B11) and 2,202 nm (B12), and one 60-m resolution band: 443 nm (B1).

## 3 Methods

### 3.1 Chlorophyll-a retrieval

In this study, a Deep Belief Network (DBN) model was utilized to retrieve Chl-a. DBN was proposed by Geoffrey Hinton in 2009 (Hinton, 2009). It is a generative model consisting of multiple layers of latent variables, constructed by several sequentially stacking Restricted Boltzmann Machines (RBMs; Mohamed et al., 2011). Each RBM undergoes layer-by-layer unsupervised pre-training, followed by supervised fine-tuning of the entire network using the backpropagation algorithm (Hua et al., 2015). DBN has found widespread applications in remote sensing retrieval (Yuan et al., 2020), such as water quality parameters (Sagan et al., 2020), leaf area index (Sun et al., 2021), and soil moisture (Yang et al., 2021). The training of the DBN in this study was conducted using an 8-layer network structure with 64 neurons in each layer and a total of 1,000 training iterations.

**Figure 2:**
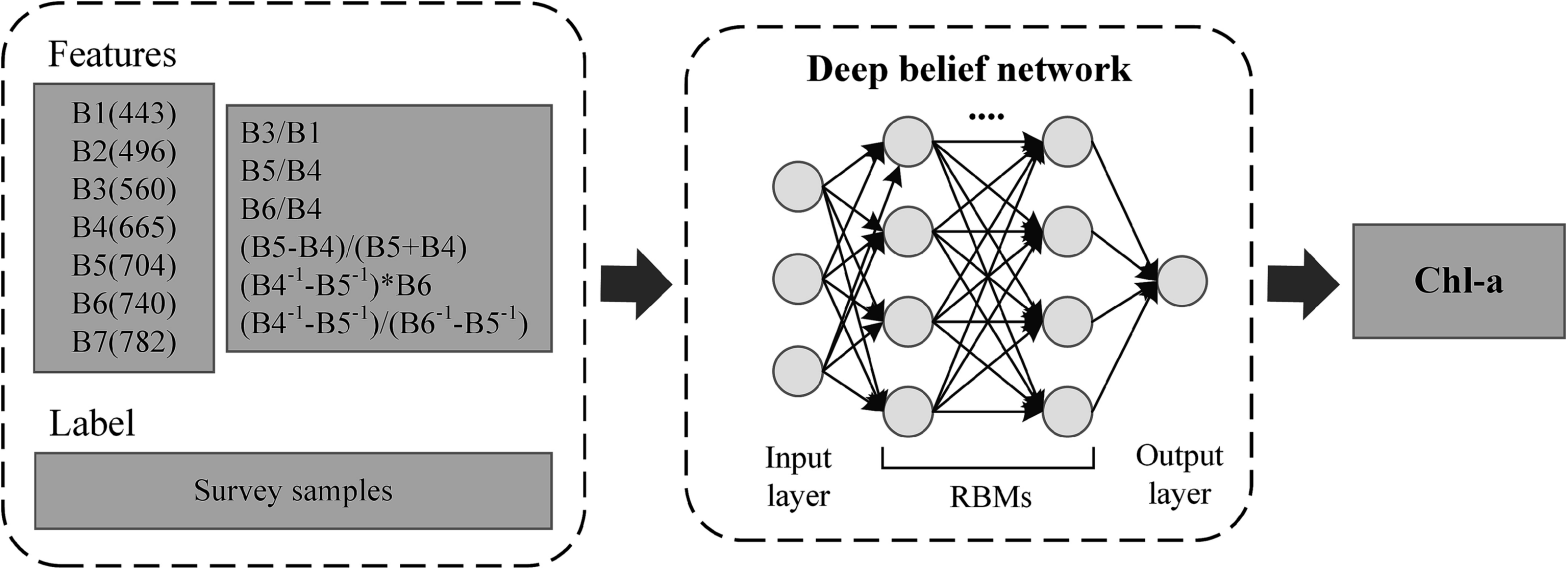
Schematic diagram of the main process for estimating chlorophyll-a concentration (Chl-a) using a deep belief network (DBN) model.

To fit the relationship between the *in situ* Chl-a and reflectance spectra (termed as $R_{rs}\left(\lambda \right)$ , λ refers to wavelength) of MSI, 80% of the samples (*N* = 227) were designated as the training dataset and input into the DBN model, while the remaining 20% (*N* = 56) were reserved as the testing dataset for assessing the model’s accuracy. The DBN model utilized in this study consists of 2 hidden layers, each with 128 neurons, and was trained for 500 epochs. The model used the *sigmoid* activation function, cross-entropy as the loss function, and stochastic gradient descent as the optimizer. The initial seven bands of MSI, with bandwidths ranging from 443 to 782 nm, were employed as input features (Li et al., 2021; Pahlevan et al., 2020). To further strengthen the robustness of the developed model, we included six band combinations derived from previously established high-performance Chl-a retrieval algorithms (Table 1). The bands specified in the reference model were substituted with the corresponding bands from the MSI. The output was the Chl-a (Figure 2).

**Table 1:** The band combinations used in this study and their reference indexes.

Band combination used	Reference band combination	References
B3560B1443	Rrs562Rrs483	Ha et al. (2017)
B5705B4665	Rrs709Rrs665	Duan et al. (2012); Gurlin et al. (2011)
B6740B4665	Rrs743~753Rrs662~672	Gitelson et al. (2008)
$\frac{B5\left(705\right)-B4\left(665\right)}{B5\left(705\right)+B4\left(665\right)}$	$\frac{R_{rs}\left(709\right)-R_{rs}\left(665\right)}{R_{rs}\left(709\right)+R_{rs}\left(665\right)}$	Mishra and Mishra, 2012, Gitelson et al. (2008)
$\left(B4{\left(665\right)}^{-1}-B5{\left(704\right)}^{-1}\right)*B6\left(740\right)$	$\left(R_{rs}{\left(\sim 670\right)}^{-1}-R_{rs}{\left(\sim 710\right)}^{-1}\right)*R_{rs}\left(\sim 750\right)$	Gitelson et al. (2011)
$\frac{B4{\left(665\right)}^{-1}-B5{\left(704\right)}^{-1}}{B6{\left(740\right)}^{-1}-B5{\left(704\right)}^{-1}}$	$\frac{R_{rs}{\left(665\right)}^{-1}-R_{rs}{\left(704\right)}^{-1}}{R_{rs}{\left(740\right)}^{-1}-R_{rs}{\left(704\right)}^{-1}}$	Yang et al. (2010)

### 3.2 Water sampling path planning

#### 3.2.1 A* algorithm

The A* algorithm, first published in 1968 by Peter Hart, Nils Nilsson, and Bertram Raphael from the Stanford Research Institute, is regarded as an extension of Dijkstra’s algorithm (Hart et al., 1968). It is a prominent heuristic search algorithm in the domain of artificial intelligence. The cost function of A* algorithm is formulated as Equation 1


(1) $$f\left(n\right)=g\left(n\right)+h\left(n\right)$$

where $g\left(n\right)$ denotes the cost from the initial node to node *n*, $h\left(n\right)$ represents the estimated cost from node *n* to the target node, i.e., heuristic function, and $f\left(n\right)$ equals to the sum of $g\left(n\right)$ and $h\left(n\right)$ (Hart et al., 1968). The selection of the cost function is heavily influenced by the specific scenario.

The heuristic function $h\left(n\right)$ has a profound impact on the performance of the A* algorithm. If $h\left(n\right)$ is always less than or equal to the cost from node *n* to the target node, the A* algorithm guarantees finding the shortest path. However, a smaller value of $h\left(n\right)$ means the algorithm will explore more nodes, leading to a slower performance. If $h\left(n\right)$ is greater than the actual cost from node *n* to the target node, the A* algorithm may not guarantee finding the shortest path, but it will be efficient. Hence, through modifying the heuristic function, we can regulate the algorithm’s speed and accuracy. In certain instances, instead of seeking the shortest path, we may prioritize finding a path as quickly as possible.

For grid-based graphs, the heuristic function can utilize Manhattan distance, diagonal distance, and Euclidean distance. In this study, the eight-neighborhood node expansion method was employed in a raster map, with the Euclidean distance from the current node to the target node as the heuristic function. The $h\left(n\right)$ is formulated as Equation 2


(2) $$h\left(n\right)=\sqrt[{2}]{{\left(x_{t}-x_{n}\right)}^{2}+{\left(y_{t}-y_{n}\right)}^{2}}$$

where $x_{t}$ and $y_{t}$ denote the horizontal and vertical coordinates of the target node, respectively, whereas $x_{n}$ and $y_{n}$ represent the coordinates of the current node *n*.

#### 3.2.2 The modified a* algorithm for water sampling path planning

**Figure 3:**
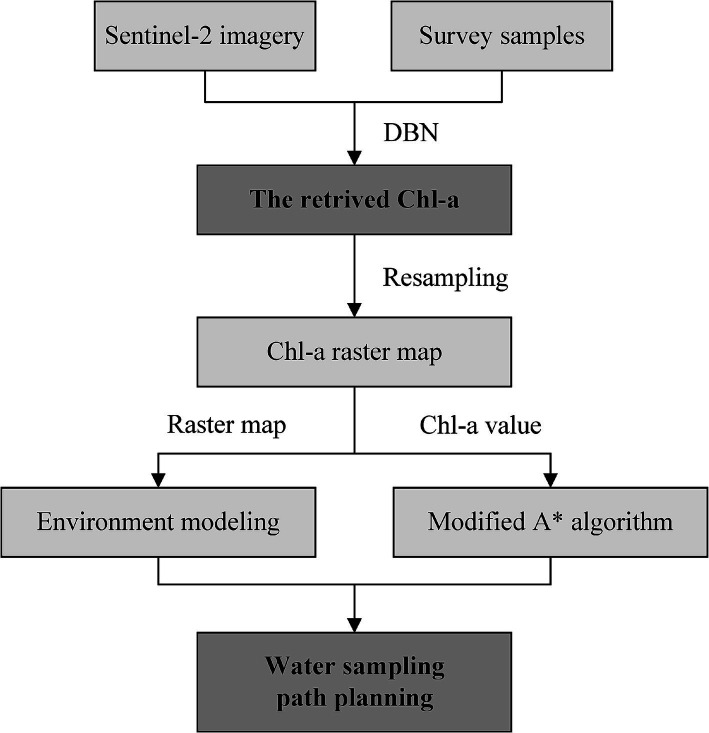
Technology roadmap of water sampling path planning for unmanned surface vehicles (USVs).

Environmental modeling is the foundation of USV path planning. In this study, Chl-a retrieved in Section 3.1 is resampled to a 200 m-resolution raster map. The Chl-a value is treated as a property of the grid. The grid with a Chl-a value was assigned 1, while the grid with null value was assigned 0, serving as an obstacle. All the grids along a USV route are considered as sampling points. By reducing the movements of USVs to eight possible directions, the trajectory is decomposed into individual movements, each of which will be recorded. The actual grid coordinates are used as unique identifiers for each grid position, allowing the planned path to be used directly for real-world sampling. The technology roadmap of water sampling path planning for USVs is demonstrated in Figure 3.

This modified A* algorithm necessitates a route that is as brief as possible, while ensuring the collected samples are representative. To ensure the sampling representativeness, it is crucial to ensure that the average Chl-a of the collected samples closely approximates the average concentration of the entire water body. Simultaneously, maintaining a gradient in Chl-a among the collected samples is also necessary. This means that Chl-a of the collected samples should not be limited to a specific concentration range, but should instead exhibit maximum variance.

In order to achieve the above purpose, the retrieved Chl-a and path length were combined as the cost function of A* algorithm, instead of only considering the path length. The key steps of the A* algorithm involve computing the movement cost from the current node to its eight neighboring nodes, and the node with the lowest cost is then removed from the OPEN_list (a list that contains all the nodes waiting for expansion) and added to the CLOSE_list (a list that contains all the nodes that have already been expanded). The improved cost function is Equation 3


$$F\left(n\right)$$

(3) $$F\left(n\right)=f\left(n\right)*Q$$

$$Q=\exp\left(-k*|Chl–a-\bar{Chl–a}|\right)$$

where $f\left(n\right)$ is the cost function only considering the path length, *Q* is called the sampling coefficient, Chl–a and $\bar{Chl–a}$ is Chl-a of an eight neighboring node and the average Chl-a of the entire water body, respectively, and k∈[0,+∞) is called the adjustment coefficient.

The adjustment coefficient *k* holds a pivotal position in the modified A* algorithm for water sampling path planning. By adjusting the value of *k*, we can modify the influence of Chl-a’s spatial distribution on the routing of USVs. As *k* increases, the algorithm plays more emphasis on the Chl-a’s spatial distribution. Thus, USV is more likely to navigate towards nodes where the Chl-a deviates from the average concentration of the entire water body ( $\bar{Chl–a}$ ). Conversely, a smaller *k* implies a greater influence of path length on route selection, with USVs more likely to choose a shorter route. In the extreme case, when the coefficient *k* equals to zero, the modified A* algorithm degenerates into a classic A* algorithm.

To validate the effectiveness of this algorithm, we recorded the path length, as well as the mean and variance of Chl-a in the collected samples, and analyzed their change trend as coefficient *k* increases. We also performed a one-way analysis of variance (ANOVA) on Chl-a of collected samples under different *k* values to assess whether the coefficient *k* has a significant effect on the variability of the collected samples.

## 4 Results

### 4.1 Chlorophyll-a retrieval

**Figure 4:**
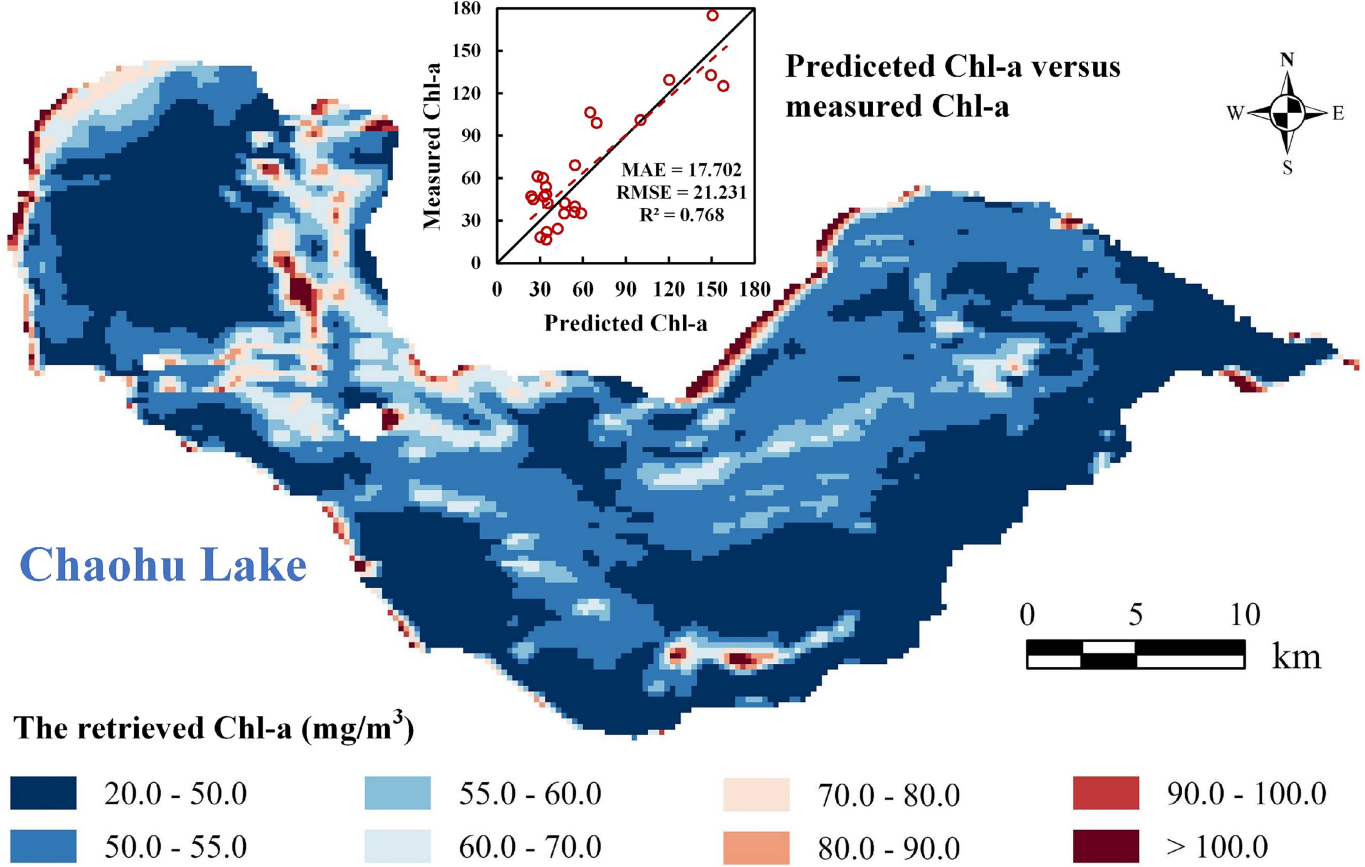
The retrieved Chl-a of Chaohu Lake in June 10, 2023 and the scatter plot of predicted and measured Chl-a.

The statistical metrics obtained from the training and testing dataset indicate good performance for the DBN model, with an R^2^ of 0.883 and 0.821, respectively. The retrieved Chl-a of Chaohu Lake in June 10, 2023 is demonstrated in Figure 4 after resampling to 200-m resolution. The retrieval results reveal that Chl-a is higher in the shoreline of Chaohu Lake and the eastern coast of its islands, usually exceeding 100 mg/m^3^, and even achieving 180 mg/m^3^. Except for these areas, the majority of regions exhibit low Chl-a, not exceeding 50 mg/m^3^. Moreover, Chl-a in the western part of Chaohu Lake is higher compared to the eastern part. This is primarily due to the proximity of the western part of Chaohu Lake to urban areas, where domestic and industrial wastewater is discharged.

The measured Chl-a values at 49 points on June 10, 2023, are compared with the retrieval values, and the resulting scatter plot is shown in Figure 4. The mean relative error (MAE), root mean square error (RMSE) and R^2^ are 17.702, 21.231, and 0.768, respectively. This indicates that the retrieved Chl-a demonstrates high accuracy, providing a robust foundation for subsequent path planning. Based on the resampled Chl-a raster map, we completed the environmental modeling for Chaohu Lake, as demonstrated in Supplementary Figure S1 online.

### 4.2 Water sampling path planning

**Figure 5:**
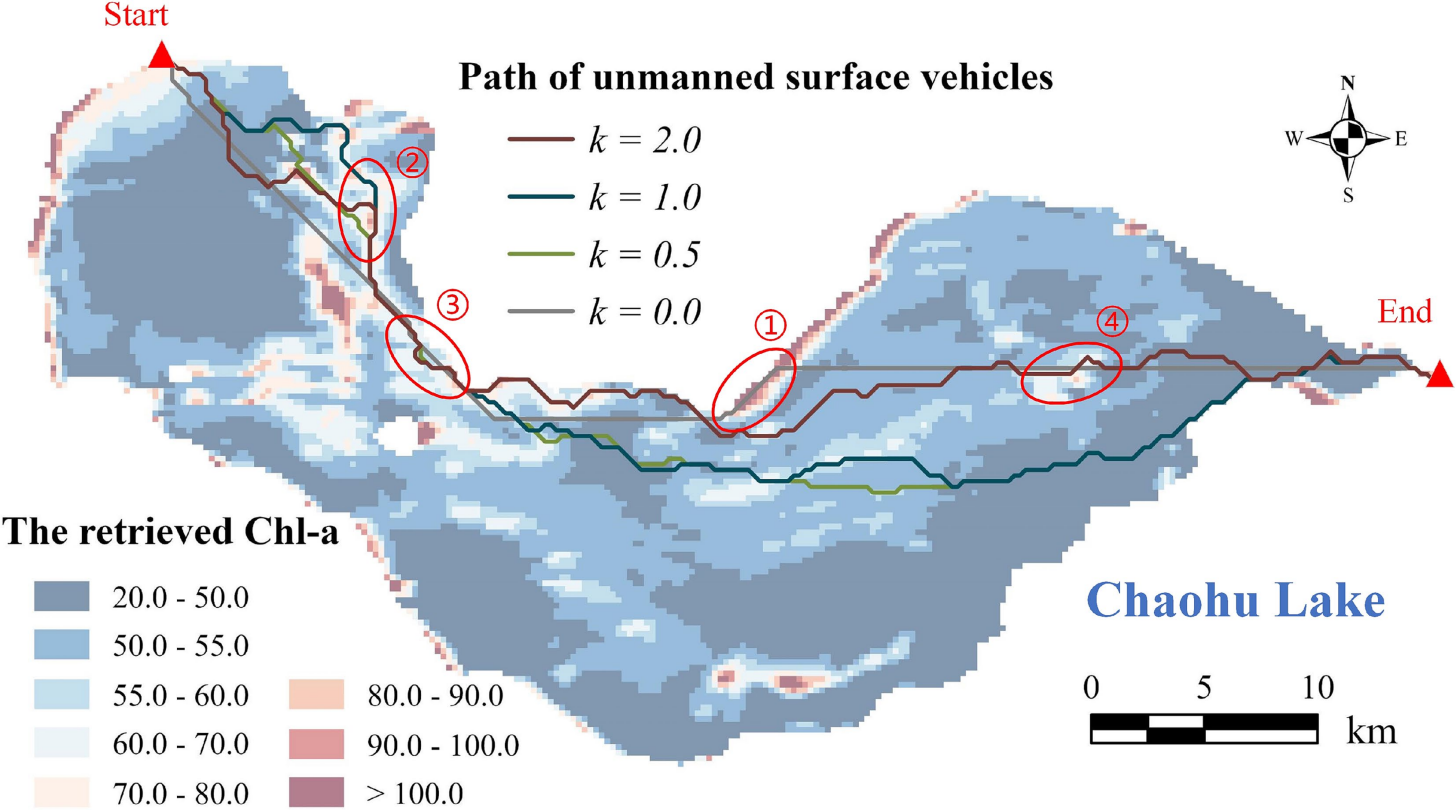
The calculated USV routes based on the modified A* algorithm. The two red triangles represent the starting and ending point, respectively.

By adjusting the coefficient *k* from 0.0 to 2.0, various USV routes are derived (Figure 5; Supplementary Figure S2). The grey route (*k* = 0) represents the shortest trajectory, with the path length of 257.17 grid units, where the spatial resolution of the raster Chl-a is 200 m. The routes depicted in green, cyan, and maroon correspond to *k* values of 0.5, 1.0, and 2.0, respectively, with path lengths of 282.18, 296.18, and 299.08 grid units accordingly.

**Figure 6:**
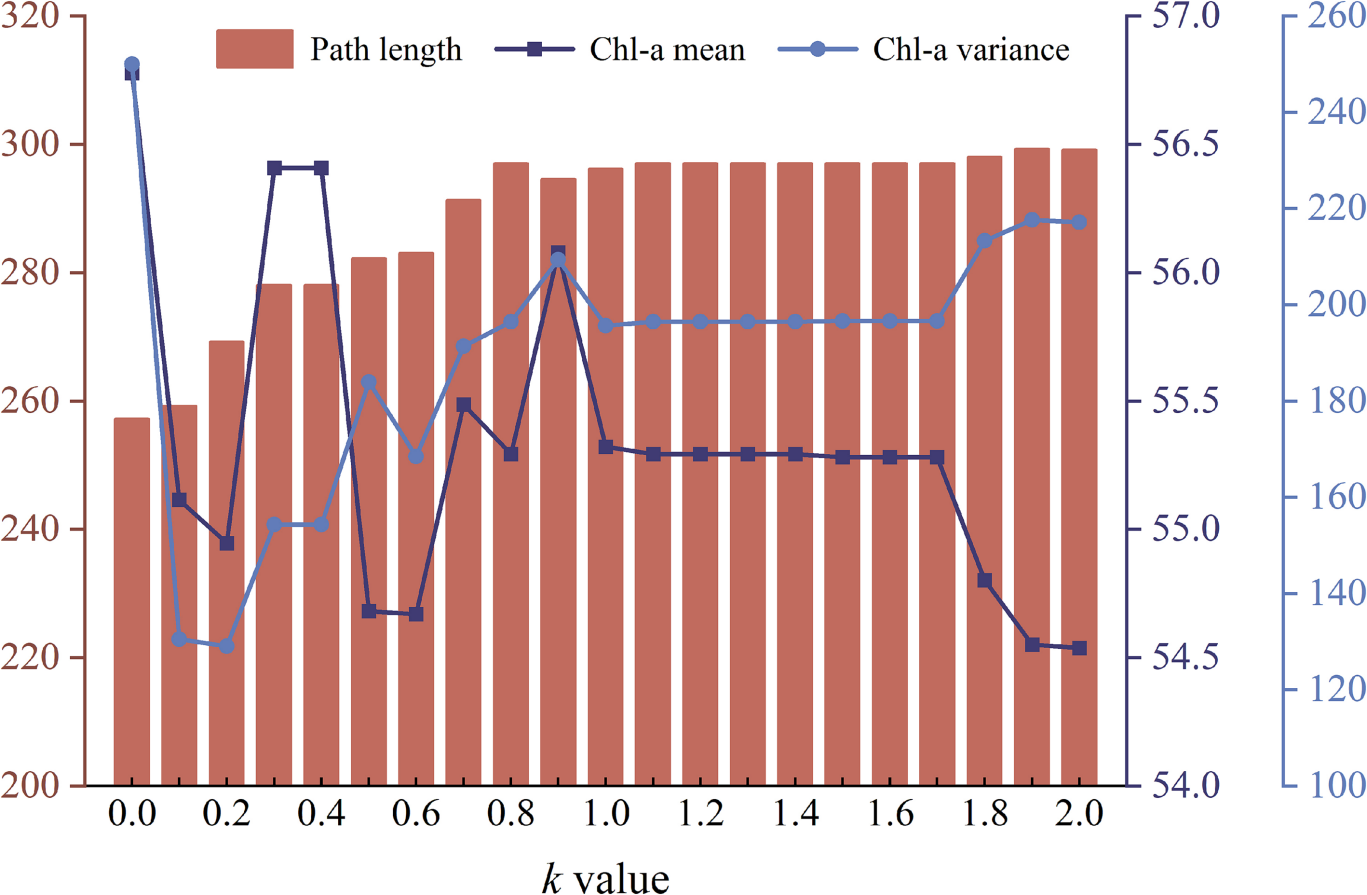
The graph of changes in path length, mean and variance of Chl-a for different routes obtained by the modified A* algorithm as the coefficient *k* increases from 0.0 to 2.0.

As the coefficient *k* increases, the modified A* algorithm demonstrates a decreasing emphasis on path length but a growing emphasis on the Chl-a’s spatial distribution (Figure 6). The path length gradually increases from 257.17 (*k* = 0) to 297.00 grid units (*k* = 0.8), until reaching a plateau between 297.00 and 299.08 grid units (*k* = 2.0). The average Chl-a of the collected samples exhibits a fluctuating decline from 56.78 to 54.54 mg/m^3^, gradually approaching the mean concentration of the entire water body ( $\bar{Chl–a}$ = 53.70 mg/m^3^). The variance of Chl-a for the collected samples increases from 130.51 (*k* = 0.1) to 217.13 (*k* = 2.0). When *k* = 0, the variance of Chl-a is extremely high (Figure 6), because the route travels through an area with exceptionally high concentration (Figure 5 ①).

**Figure 7:**
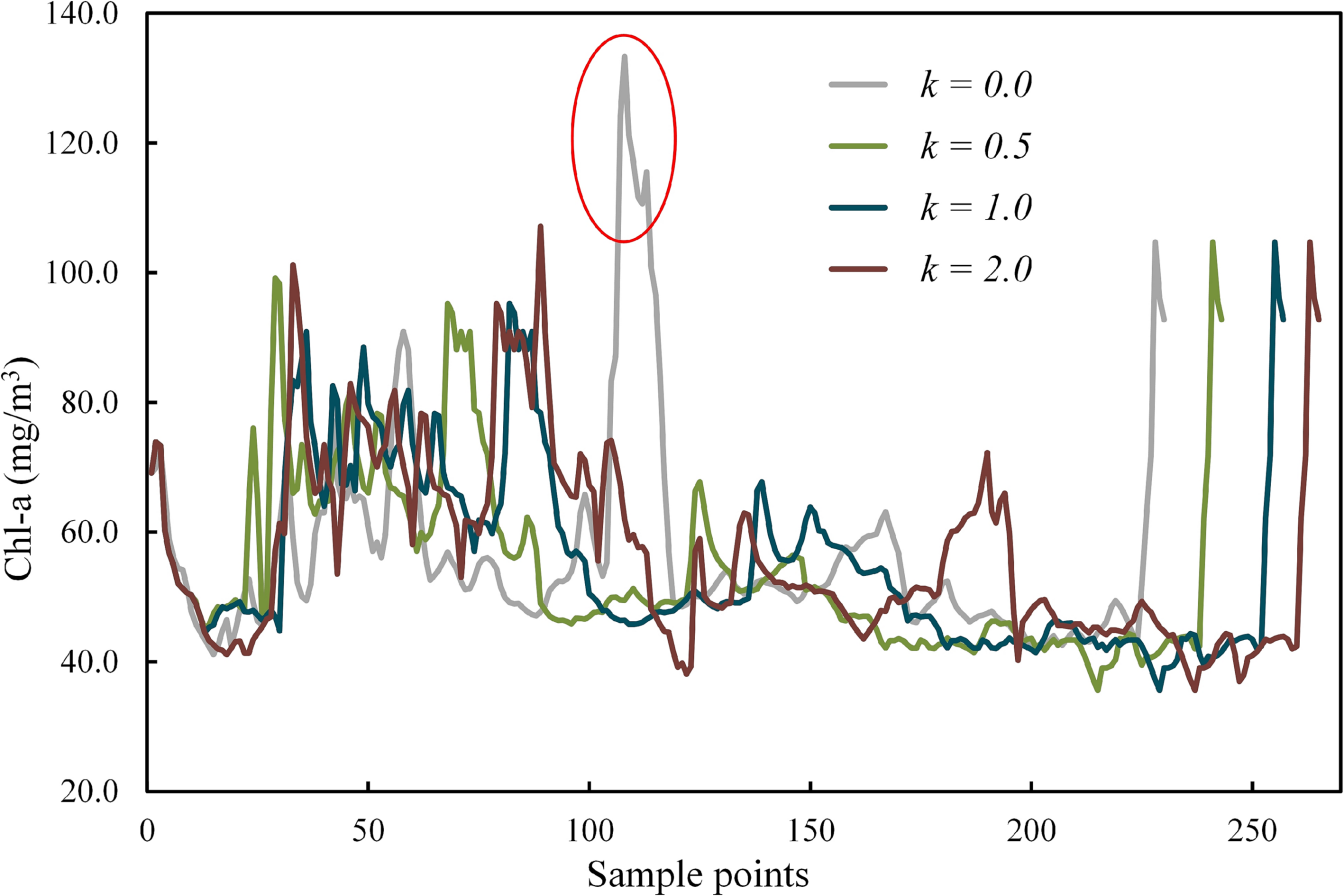
Chl-a of collected samples for routes with different coefficient k obtained by the modified A* algorithm.

Chl-a of all collected samples along USV routes with different coefficient *k* is represented in Figure 7. The peak in the gray line (*k* = 0) marked with a red circle indicates the area with unusually high Chl-a (Figure 5 ①). As the coefficient *k* rises from 0.5 to 2.0, the line chart of Chl-a exhibits increasingly pronounced fluctuations, with corresponding variances of 183.97, 195.67, and 217.13, respectively. This phenomenon can also be observed in Figure 5 ②–④, where the three colored routes, as opposed to the grey route (the shortest route), tend to pass through areas where Chl-a deviates from the overall mean concentration. The results of one-way ANOVA revealed that coefficient *k* has a significant effect on the variability of Chl-a in the collected samples, with an *F* value of 1.781 exceeding the critical value of 1.573, and a *p* value of 0.017 (<0.05).

## 5 Discussion

### 5.1 Comparisons with previous studies

This study proposed a modified A* algorithm that takes into account not only the path length, but also the sampling representativeness in the water quality sampling path planning of USVs. The verification results showed that the proposed algorithm was effective in real-world conditions and achieved the trade-off between the representativeness of sampling points and the path length. As the coefficient *k* increased, the representativeness of the collected samples enhanced, but this came at the cost of increased path length, and vice versa.

This study differs significantly from previous researches, which focused primarily on factors such as path length, time consumption, path smoothness, and collision avoidance. Until now, there have been no observed studies on USV-based water quality sampling path planning that consider the representativeness of collected samples. Table 2 lists several typical studies on USV path planning, including studies on water quality sampling path planning of USVs. Researches on path planning with a single target point (referred to here as single-goal path planning) can be broadly categorized into global, local, and hybrid algorithms. In all three types of studies, path design typically takes into account both path length and time consumption, while some studies also consider path smoothness. Global path planning also focuses on global obstacle avoidance, whereas local path planning usually emphasizes dynamic obstacle avoidance. Hybrid algorithms typically take into account all the aforementioned four factors. For instance, Yu et al. (2019) introduced a hybrid approach that combined A* and artificial potential field. This method took into account the four factors and demonstrated effectiveness in surface water environment with unknown districts. Multi-goal path planning algorithms typically focus on path length and time consumption, with few studies also considering path smoothness, such as Zhao et al. (2023) and Yu et al. (2024).

**Table 2:** Factors in consideration for previous studies on USV path planning.

Single-/Multi-goal path planning		Algorithm	Factors in consideration				References
			Path length	Time consumption	Path smoothness	Dynamic obstacle avoidance	
Single-goal	Global	A* algorithm	√	√	√		Song et al. (2019)
		Constrained A* algorithm	√	√		√	Singh et al. (2018)
		Complete coverage neural network	√	√	√	√	Xu et al. (2021)
		Finite Angle A* algorithm	√	√	√		Yang et al. (2015)
	Local	Constrained fast marching	√	√		√	Liu and Bucknall, 2015
		Locking sweeping method	√	√	√	√	Su et al. (2023)
		Improved artificial potential field	√	√		√	Song et al. (2018)
		Model predictive artificial potential field	√	√	√	√	He et al. (2023)
	Hybrid	A* + Artificial potential filed	√	√	√	√	Yu et al. (2019)
		B-Spline data frame + Particle swarm optimization	√	√	√	√	MahmoudZadeh et al. (2022)
		Improved A* + Artificial potential field	√	√	√	√	Sang et al. (2021)
		Non-uniform Theta* + Improved dynamic window approach	√	√	√	√	Han et al. (2022)
Multi-goal		Greedy Partheno Genetic Algorithm	√	√	√		Zhao et al. (2023)
		Genetic algorithm based on improved crossover operators	√	√			Chen et al. (2021)
		Self-organizing map + Artificial repulsive force field	√	√			Liu and Bucknall, 2018
		D*Lite algorithm + Dubins search tree algorithm + Reeds-Shepp curves	√	√	√		Yu et al. (2024)

Another distinguishing aspect of our path planning research with current studies is its integration of remote sensing technology, which is rarely seen in current USV path planning field. Although, some recent studies have employed satellite imagery for environmental modelling in USV path planning, demonstrating the practicality of their algorithms in the real world (Yang et al., 2015; Su et al., 2023; Song et al., 2019), these studies fail to fully harness the information contained within the imagery. Our study, for instance, utilized Chl-a values retrieved from remote sensing imagery as prior knowledge to facilitate the collection of representative samples.

### 5.2 Possible factors influencing the adjustment amplitude of coefficient *k*

As mentioned above, the modified A* algorithm can alter the emphasis placed on path length and Chl-a’s spatial distribution by adjusting the coefficient *k*. Therefore, after obtaining the retrieved Chl-a and determining the starting and ending points of USVs, our only approach is to incrementally increase the *k* value to achieve the optimal routing. However, we still do not know the appropriate adjustment amplitude for a specific water body. The distance between the starting and ending points and the data distribution of Chl-a are two potential factors influencing the adjustment amplitude of coefficient *k*.

#### 5.2.1 The distance between the starting and ending points

The distance between the starting and end points of USVs has a significant impact the adjustment amplitude of coefficient *k*, with the adjustment amplitude of *k* generally increasing as the distance between these two points decreases. In practical experiments, it is common for the starting and end points of USVs to be very close or even coinciding. In this case, selecting routes with more representative samples, as opposed to the shortest route, will lead to a significant increase in path length. Therefore, after calculating the total cost, the modified A* algorithm tends to select a route with a shorter length rather than one with more representative samples. Thus, to address this issue, increasing the value of coefficient *k* is essential to place greater emphasis on the spatial distribution of Chl-a within the algorithm. This adjustment will compel the algorithm to overcome the cost associated with increased path length and to consider the spatial distribution of Chl-a.

**Figure 8:**
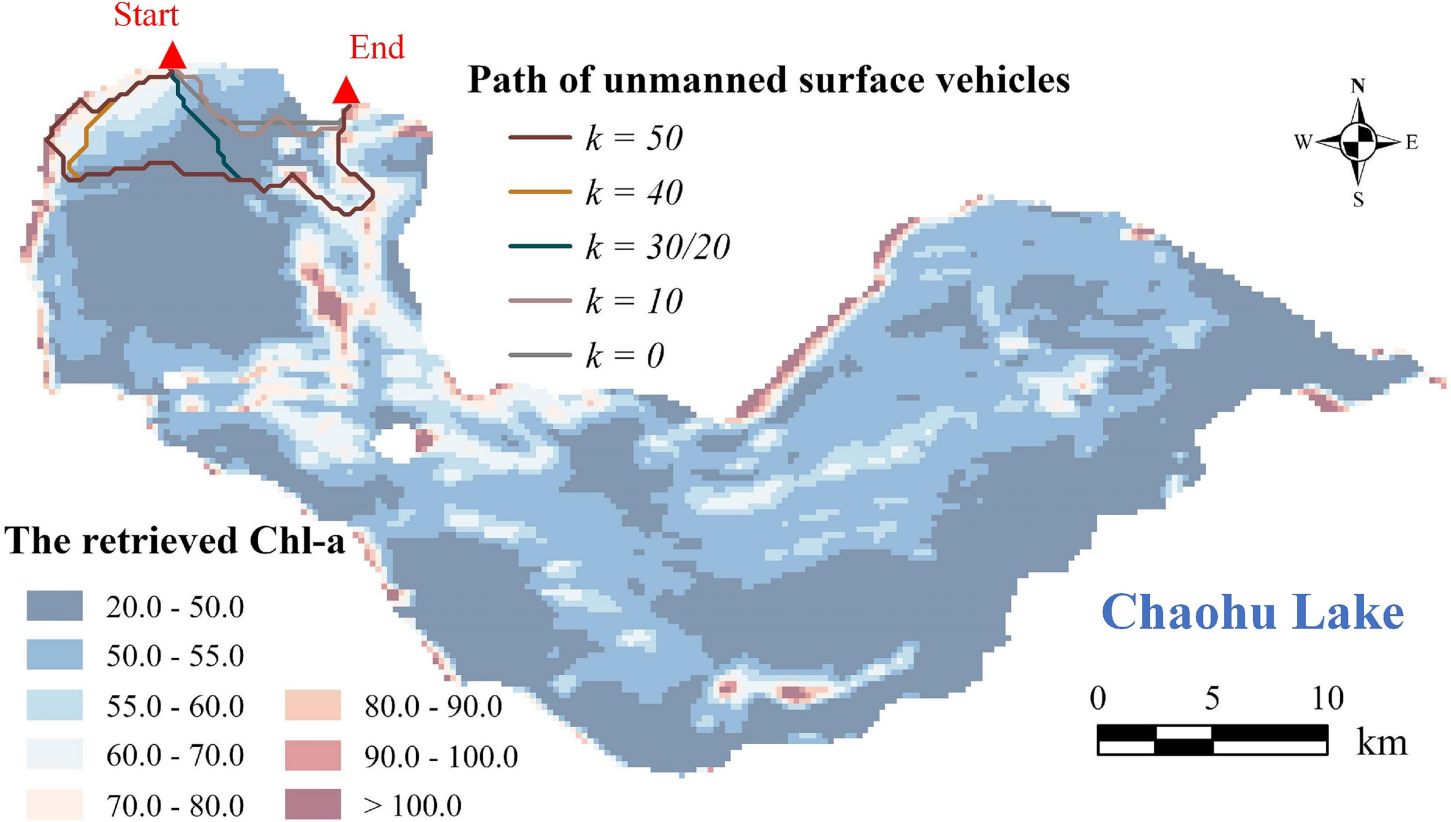
USV paths based on the modified A* algorithm when the starting and ending points are in close proximity.

Figure 8 suggests that when the starting and ending points are in close proximity, the adjustment amplitude of coefficient *k* is 20 times greater in order to significantly alter the routes compared to when these two points are at a greater distance (Figure 5). Additionally, the routes calculated with an adjustment amplitude of 1.0 are depicted in Supplementary Figure S3. It is evident that the routes generated by the modified A* algorithm (*k* > 0) primarily focus on path length rather than the Chl-a’s spatial distribution, resulting in slight variations compared to the shortest path (*k* = 0).

#### 5.2.2 The data distribution of Chl-a

**Figure 9:**
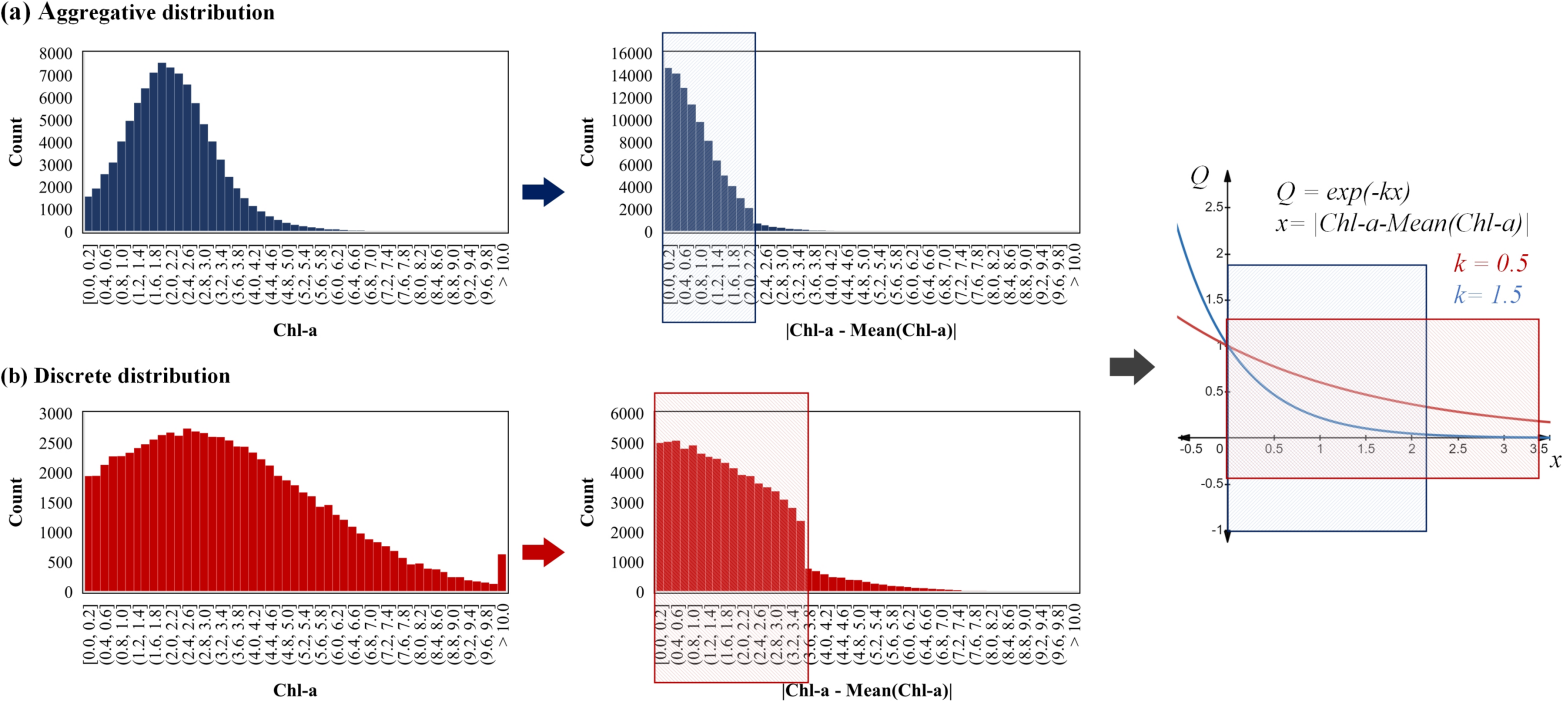
The influence of Chl-a data’s distribution to the adjustment amplitude of coefficient *k*.

As the aggregation degree of Chl-a’s data distribution increases, a larger adjustment amplitude of coefficient *k* is typically required. For the sampling coefficient $Q=\exp\left(-kx\right)$ , the convergence rate to zero accelerates with an increase of the adjustment coefficient *k* (*k* ≥ 0). As the distribution of Chl-a data becomes more aggregated, the value of Chl–a−Chl–a¯ will tend to concentrate to zero (Figure 9). This necessitates that the function $Q=\exp\left(-kx\right)$ converges rapidly, resulting in a broadened distribution range of *Q* values (avoiding excessive concentration to 1). The expansion of *Q* will further affect the distribution of the cost function *F(n)* (Equation 3), thereby amplifying the influence of Chl-a’s spatial distribution on route regulation.

The two datasets presented in Figure 9 are used as a reference. Dataset A, which is more aggregated with a smaller range of Chl–a−Chl–a¯ is more suitable for the function $Q=\exp\left(-1.5x\right)$ with faster convergence speed (colored with blue). Conversely, Dataset B better suited for the function $Q=\exp\left(-0.5x\right)$ with slower convergence speed (colored with red). However, if the function $Q=\exp\left(-0.5x\right)$ is employed for the aggregated dataset A, the obtained *Q* value will tend to concentrate to 1. Conversely, if the function $Q=\exp\left(-1.5x\right)$ is employed for dataset B, the range of *Q* values obtained will be concentrated to 0. Therefore, when the aggregation degree of Chl-a’s data distribution increases, a larger adjustment amplitude of coefficient *k* is typically more appropriate.

### 5.3 Shortcomings and future work

This study proposed a modified A* algorithm that concurrently considers the spatial distribution of water quality parameters and path length. While this algorithm exhibits perfect results, it also has several shortcomings.

Firstly, the use of deep learning constrains the practicality of this approach. Actually, the algorithm proposed in this study does not necessitate high precision in the retrieval of water quality parameters, and it solely requires an approximate determination of their spatial distribution. Thus, empirical models (Woo Kim et al., 2022; Li et al., 2021; Gurlin et al., 2011; Gitelson et al., 2008) can also be effectively employed in the parameter retrieval. Moreover, the readily available Chl-a products from satellites like Sentinel-3 can also be used directly, which could provide comprehensive coverage and consistent data quality (Pahlevan et al., 2020).

Secondly, the algorithm is not applicable when the starting and ending points are completely coincident. Nevertheless, effectively addressing the routing problem can be achieved by maintaining an adequate distance between these two points and increasing the adjustment amplitude of coefficient *k*.

Thirdly, considering that water quality parameters often display a right-skewed distribution (with the distribution of retrieved Chl-a in Chaohu Lake presented in Supplementary Figure S4), it is worth further exploring whether using the median instead of the mean ( $\bar{Chl–a}$ ) would be more suitable in cases of extreme right skewness.

Fourthly, this study utilized Chl-a as the water quality parameter to test the proposed algorithm, and additional water parameters such as turbidity, colored dissolved organic matter (CDOM), and total suspended matter (TSM) could also be employed for further testing purposes.

Fifthly, the proposed modified A* algorithm is an initial version aimed at highlighting the importance of representativeness of sampling points in water sampling path planning. Future optimizations could include collision avoidance, increasing path smoothness, and improving efficiency etc.

## 6 Conclusion

To address the easily overlooked problem of representative sampling in the water quality sampling path planning of USVs, a modified A* algorithm that integrates remote sensing technique has been proposed. Specifically, the heuristic function of the A* algorithm was modified by incorporating a sampling coefficient *Q* to ensure the representativeness of collected samples. Subsequently, an adjustment coefficient *k* was introduced into the coefficient *Q* to optimize the trade-off between the representativeness of sampling points and the path length. The verification results showed that the proposed algorithm was effective in collecting more representative samples in real-world conditions. As the coefficient *k* increased, the representativeness of the collected samples enhanced, indicated by the Chl-a closely approximating the overall mean Chl-a and exhibiting a gradient distribution. However, this enhancement was also associated with increased path length, and vice versa. The distance between the starting and ending points and the data distribution of water quality parameters are two potential factors influencing the adjustment amplitude of coefficient *k*. A reduced distance between the starting and ending points or an increased degree of aggregation in the distribution of parameter data will result in a larger adjustment amplitude of coefficient *k*.

Regarding future research, Chl-a values retrieved from empirical models or Chl-a products can serve as prior knowledge for water quality sampling path planning. Besides Chl-a, water parameters like turbidity, CDOM, and TSM can be employed for algorithm testing. The proposed algorithm should also be optimized to avoid collision, increase path smoothness, and improve efficiency etc.

## Data Availability

The raw data supporting the conclusions of this article will be made available by the authors, without undue reservation.

## Footnotes

^1^ https://earthengine.google.com/
