# Supplementary material for: A modified A* algorithm combining remote sensing technique to collect representative samples from unmanned surface vehicles
Source: Front Neurorobot. 2024 Oct 22;18:1488337. doi: 10.3389/fnbot.2024.1488337 (PMC11535655; doi:10.3389/fnbot.2024.1488337)
Supplement: Supplementary file 1 [file Data_Sheet_1.DOCX]

Supplementary Material

# Supplementary Figures and Tables

## Supplementary Figure


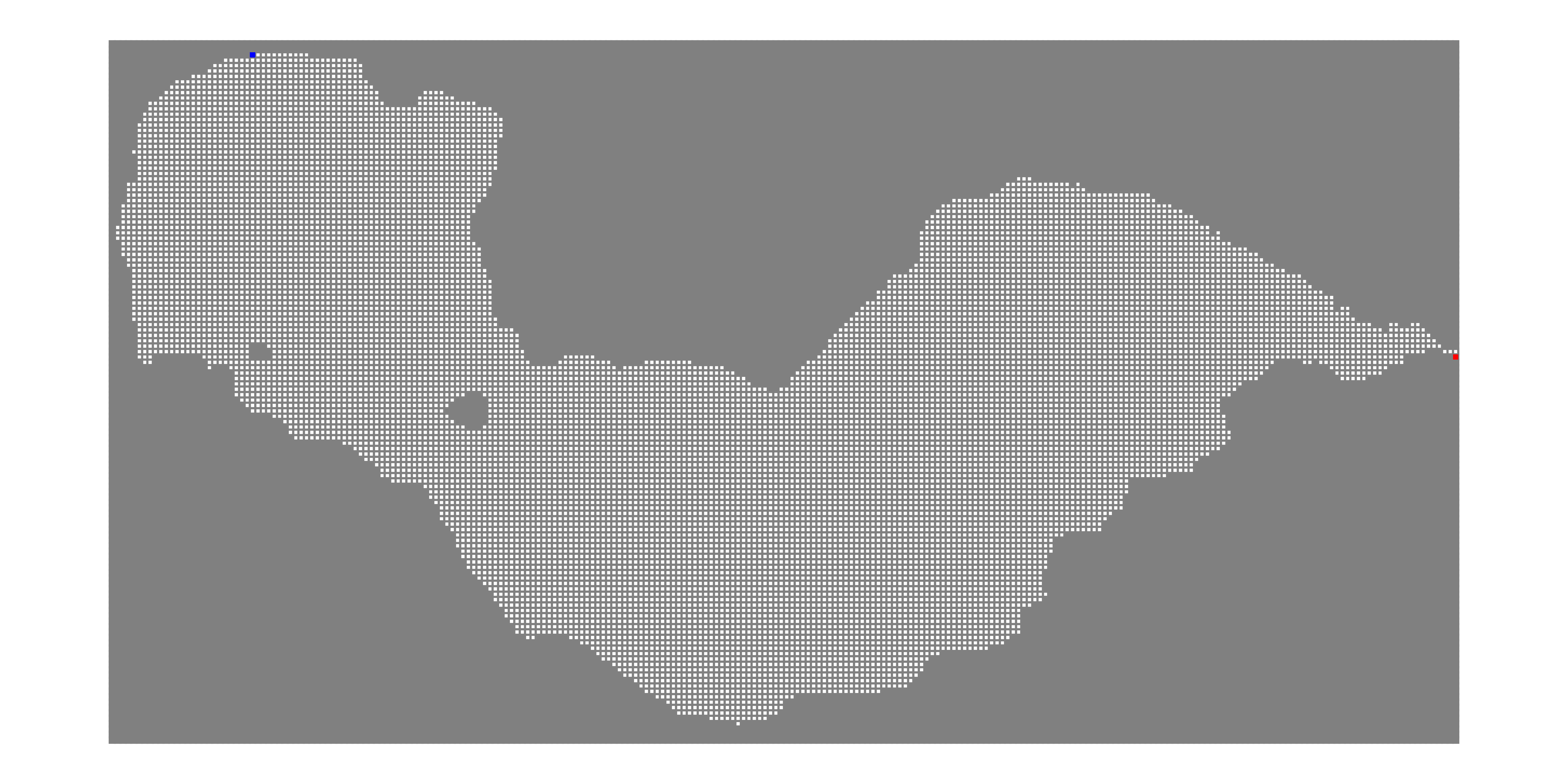


**Figure S1.** The environmental modeling of Chaohu Lake. Gray areas indicate obstacles. The blue and red grids represent the starting and ending points of unmanned surface vehicles (USVs), respectively.


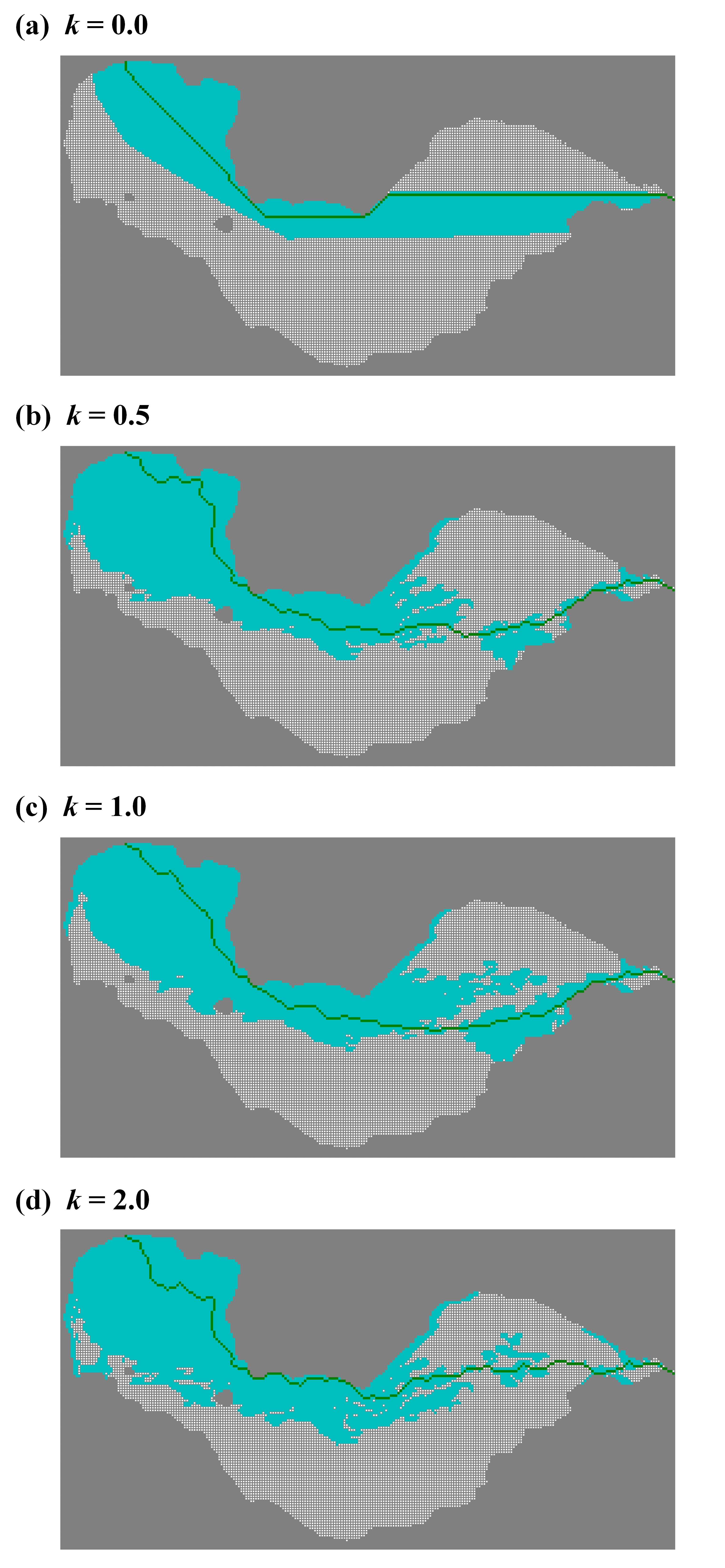


**Figure S2.** USV paths based on the modified A* algorithm. The green grid denotes the planned route, while the cyan grid indicates the search range of the algorithm.


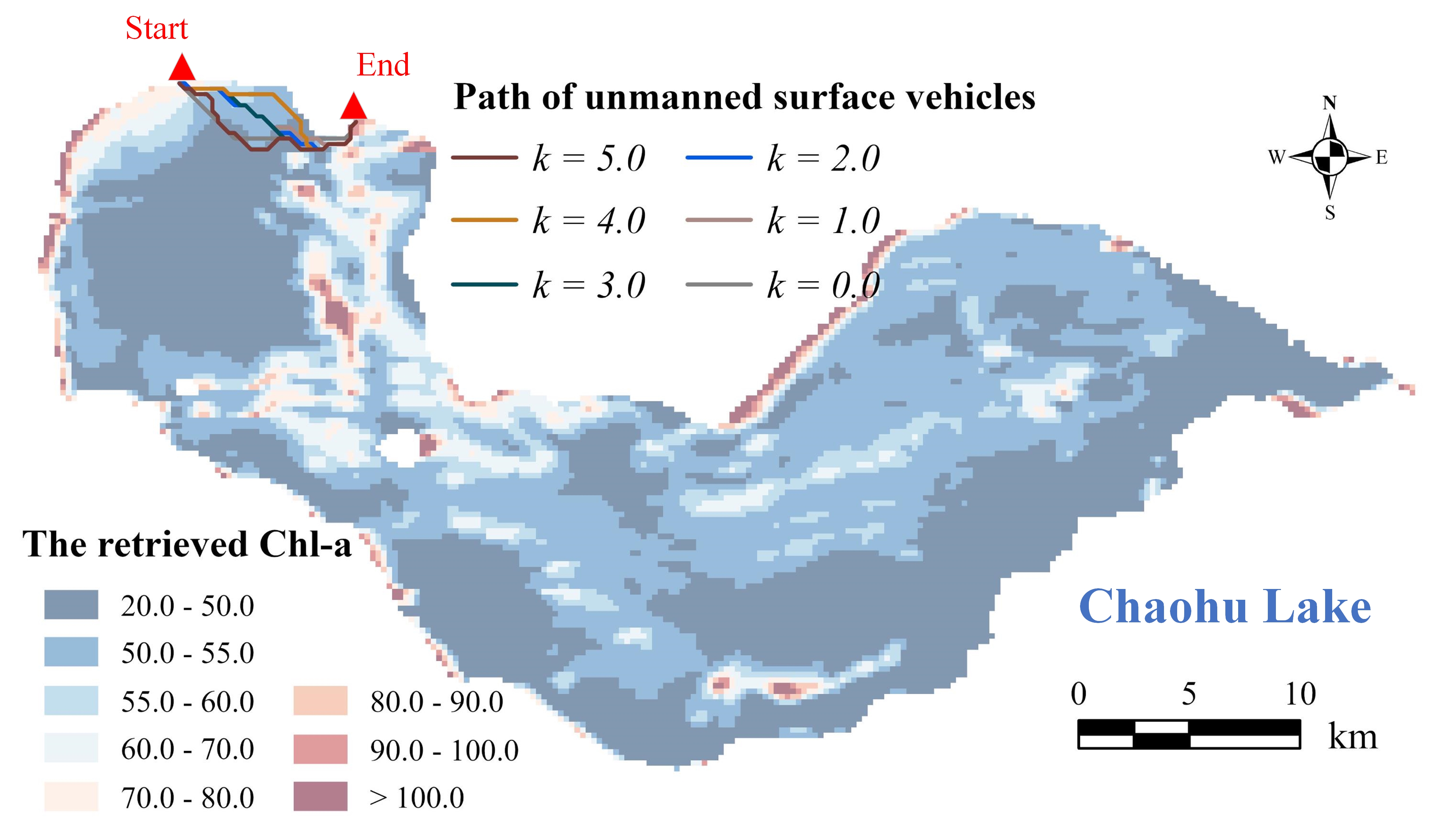


**Figure S3.** USV paths based on the modified A* algorithm when the starting and ending points are in close proximity.


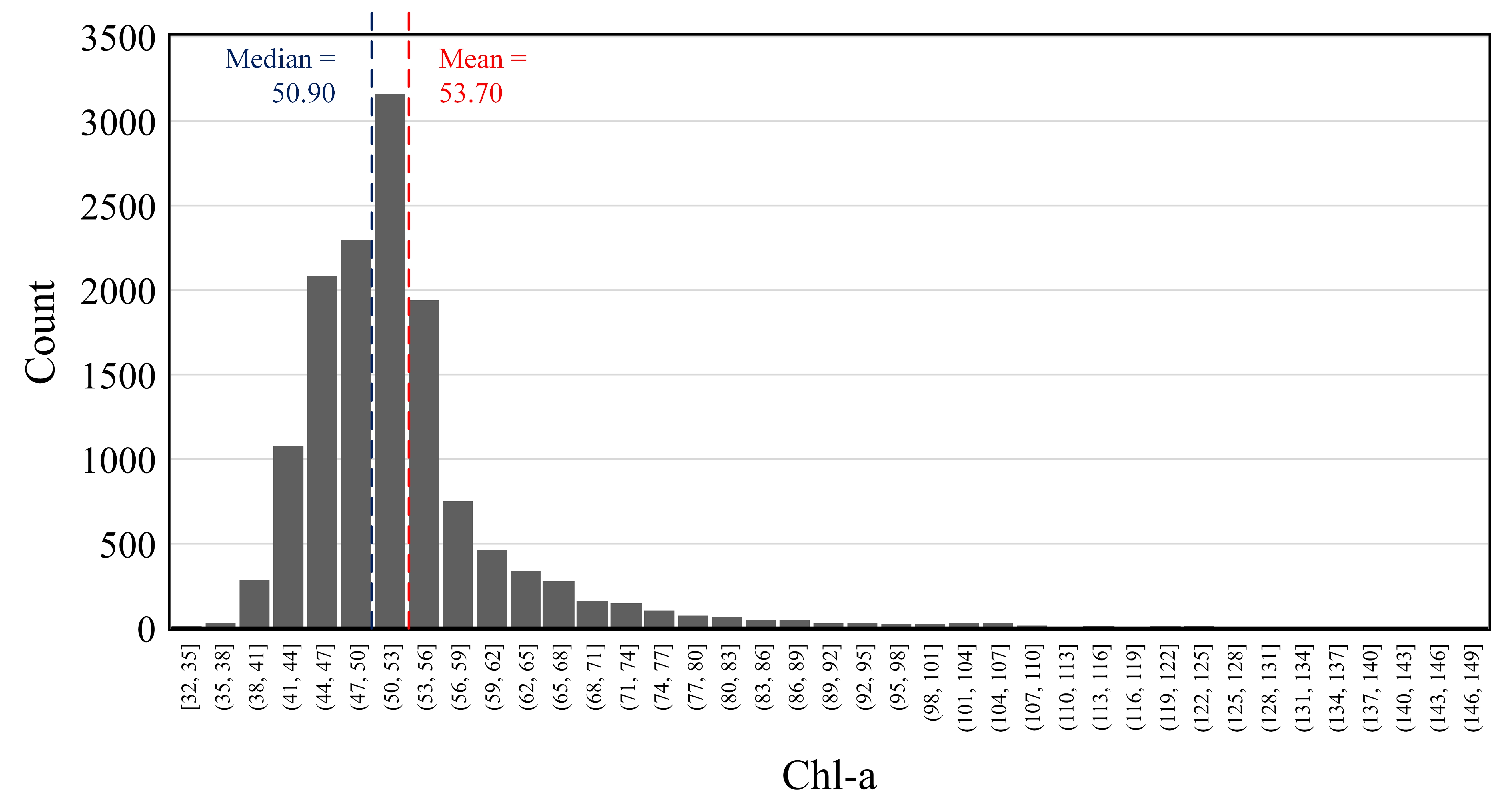


**Figure S4.** Data distribution of Chl-a (mg/m3) in Chohu Lake.
